# Primary pulmonary mucosa-associated lymphoid tissue lymphoma with amyloid light chain-type amyloidosis

**DOI:** 10.1186/s40792-019-0663-0

**Published:** 2019-06-26

**Authors:** Shuta Ohara, Kenji Tomizawa, Shigeki Shimizu, Kenichi Suda, Toshio Fujino, Akira Hamada, Takamasa Koga, Masaya Nishino, Yoshihisa Kobayashi, Katsuaki Sato, Masato Chiba, Masaki Shimoji, Toshiki Takemoto, Junichi Soh, Tetsuya Mitsudomi

**Affiliations:** 1 Division of Thoracic Surgery, Department of Surgery, Faculty of Medicine, Kindai University, 377-2 Ohno-Higashi, Osaka-Sayama, 589-8511 Japan; 2 Department of Pathology, Faculty of Medicine, Kindai University, Ohno-Higashi, Osaka-Sayama, Japan; 3 Department of Thoracic Oncology, Dana-Farber Cancer Institute, Boston, USA

**Keywords:** Lung, Mucosa-associated lymphoid tissue lymphoma, Amyloid deposition

## Abstract

**Background:**

A total of 75% of patients with Sjögren’s syndrome are complicated with pulmonary lesions, of which 12% are lymphoma and 6% are amyloid nodules; the coexistence of both is considered to be rare.

**Case presentation:**

A 67-year-old female with Sjögren’s syndrome presented with multiple pulmonary nodules on chest computed tomography. Since a definitive diagnosis by transbronchial biopsy was not obtained, wedge resection of the nodules was performed. Pathologic diagnosis revealed eosinophilic deposition that stained positive with Congo red. In addition, lymphoepithelial lesions and lymphocytic infiltration were observed. Lymphocytes with monoclonal proliferation predominantly had κ chain. Based on these findings, the nodules were diagnosed as mucosa-associated lymphoid tissue (MALT) lymphoma with amyloid deposition.

**Conclusions:**

The combination of these diseases is very rare, and this is the sixth resected case to the best of our knowledge.

## Background

Sjögren’s syndrome is an autoimmune disease characterized by chronic inflammation of systemic exocrine glands and dry symptoms owing to impaired function of the exocrine glands. Most patients with Sjögren’s syndrome have been reported to have pulmonary lesions; of these, 61% had nonspecific interstitial pneumonia, 58% had pulmonary fibrosis, 12% had bronchiolitis, 12% had malignant lymphomas including mucosa-associated lymphoid tissue (MALT) lymphoma, 6% had amyloid nodules, and 6% had atelectatic fibrosis [1]. Of those reported with MALT lymphoma, 9% had lung nodules [2]. Also, amyloidosis is a systemic disease caused by the accumulation of amyloid in the entire body. Nodular pulmonary amyloidosis (NPA) is extremely rare [3] with few reports on the association between MALT lymphoma and NPA [4–8]. Here, we report a rare case of MALT lymphoma with amyloid deposition.

## Case presentation

**Fig. 1:**
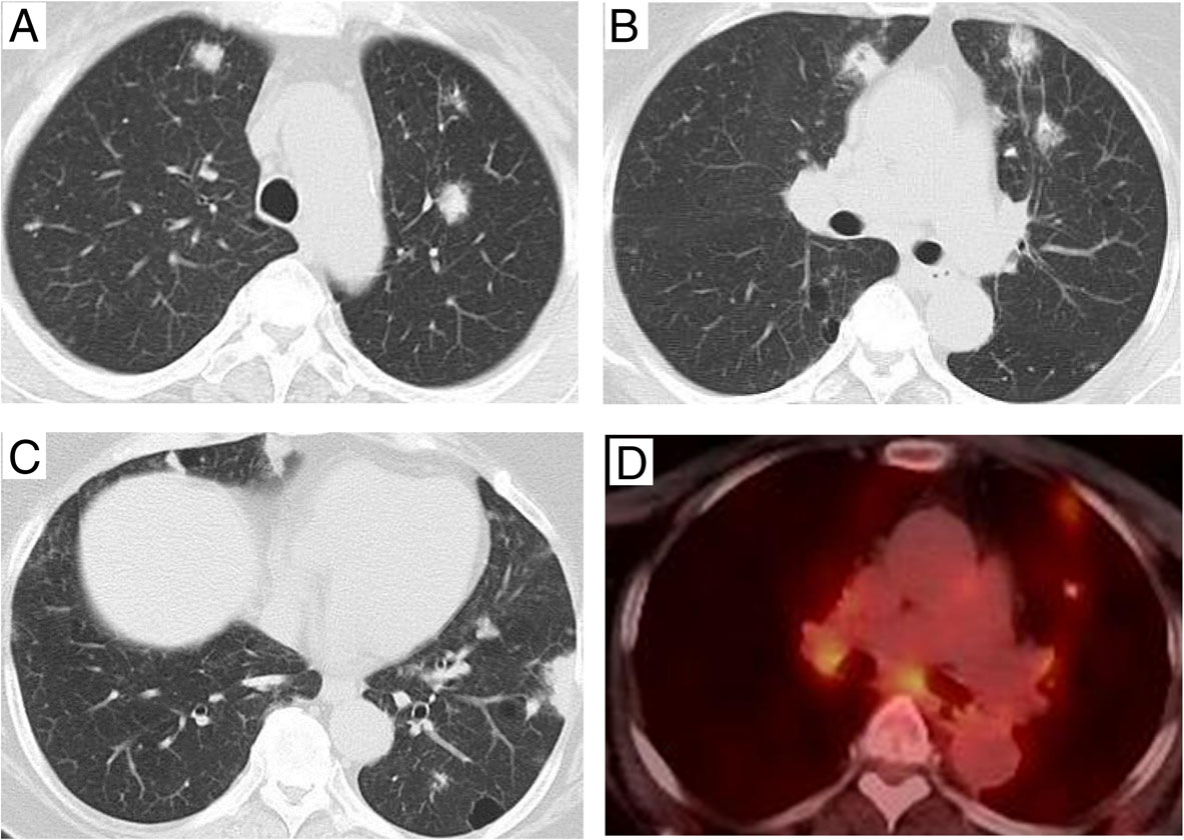
**a**–**c** Chest computed tomography showed multiple nodules in the right S3, right S7, left S1 + 2, and left S8. **d** Fluorodeoxyglucose-positron emission tomography showed FDG accumulation in pulmonary nodules to the horizontal slice as **b**

**Fig. 2:**
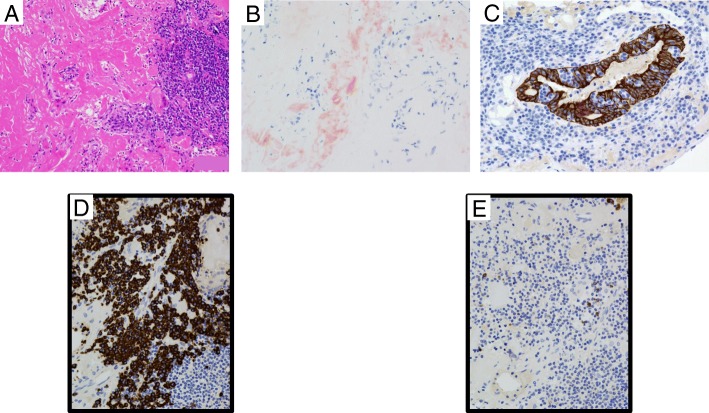
**a** Eosinophilic deposition with lymphoplasmacytic infiltration was noted. **b** The eosinophilic deposition stained positive with Congo red. **c** Immunostaining for AE1/AE3 and CD20 revealed extensive infiltration of B cells to the bronchial epithelial cells (lymphoepithelial lesion). **d**, **e** B cells with monoclonal proliferation predominantly had a κ chain by in situ hybridization (κ chain: λ chain ratio, 20:1)

A 67-year-old female with Sjögren’s syndrome was found to have multiple pulmonary nodules in both lungs on chest computed tomography (CT) (Fig. 1a–c), because she was found with multiple pulmonary nodules on chest x-ray. She had a history of hypertension and osteoporosis. No increase was observed in the levels of tumor markers, such as carcinoembryonic antigen and squamous cell carcinoma antigen. Pulmonary functions were within normal limits. Fluorodeoxyglucose-positron emission tomography (FDG-PET) showed FDG accumulation in all pulmonary nodules (Fig. 1d). A transbronchial biopsy results in alveolar tissue showing interstitial fibrous thickening and lymphoid cells infiltrations. Since it did not result in a definitive diagnosis, the patient was admitted to our department for a surgical biopsy. Because a substantial amount of tissue is essential for the diagnosis, CT-guided lung biopsy was not performed. Wedge resection was performed for pulmonary nodule located beneath the pleura of left lower lobe. The nodule was gray, elastic, and hard. Intraoperative frozen-section analysis was performed that suspected amyloid nodule with lymphoma. In a permanent pathological section, eosinophilic deposition with lymphoplasmacytic infiltration was noted (Fig. 2a). Because the eosinophilic deposition stained positive with Congo red (Fig. 2b), a diagnosis of amyloidosis was established. In addition, immunostaining for AE1/AE3 and CD20 revealed extensive infiltration of B cells to the bronchial epithelial cells (lymphoepithelial lesion) (Fig. 2c). B cells with monoclonal proliferation predominantly had a κ chain by in situ hybridization (κ chain: λ chain ratio, 20:1) (Fig. 2d, e). The findings of immunohistochemical staining were as follows: CD3 (+), CD20 (+), CD138 (+), bcl-2 (+), CD5 (−), CD10 (−), CD23 (−), CD56 (−), and cyclin D1 (−). This nodule was diagnosed as mucosa-associated lymphoid tissue (MALT) lymphoma. The postoperative course was uneventful. She was discharged on the fourth day after surgery and received chemotherapy for MALT lymphoma at another hospital.

## Discussion

The causes of amyloidosis include idiopathic, chronic inflammatory diseases, collagen diseases (such as Sjögren’s syndrome), and lymphoproliferative diseases [9]. Pulmonary amyloidosis is classified into nodular pulmonary amyloidosis (NPA), tracheobronchial amyloidosis, and alveolar septal amyloidosis. Although alveolar septal amyloidosis is associated with systemic amyloidosis, NPA and tracheobronchial amyloidosis are confined to the lung and bronchial trachea, respectively. Tracheobronchial amyloidosis is characterized by amyloid deposition in the trachea and bronchial mucosa, and typical symptoms include cough, wheezing, hemoptysis, and dyspnea [10]. In the present case, the patient did not present such symptoms as well as tracheobronchial lesions. Therefore, these pulmonary nodules were considered to be NPA. NPA is classified into AL (amyloid light chain) and AA (amyloid A) type amyloidosis; AL type is caused by immunoglobulin light chains produced by neoplastic plasma cells, and AA type is caused by chronic inflammation of autoimmune etiology. The present case showed κ type-predominant AL-type amyloids. In this case, amyloid depositions of AL type were considered to be produced by plasma cells of MALT lymphoma [4–8, 11].

To the best of our knowledge, only six patients with primary lung MALT lymphoma with amyloid deposition including ours have been reported (Table 1) [4–8]. Of these patients, two were symptomatic (dyspnea, cough, and chest pain), four had multiple lesions, and four patients had an autoimmune disorder. Five of these were diagnosed by surgical resection.

**Table 1:** Primary pulmonary mucosa-associated lymphoid tissue lymphoma with amyloid deposition

Authors	Age/sex	Symptom	Number of tumors	Comorbidity	Procedure
Fujiwara et al. [4]	60/F	None	Single	None	Right upper middle lobectomy
Satani et al. [5]	65/F	None	Multiple	RA	Left upper lobectomy
Nakamura et al. [6]	53/F	Cough, chest pain	Single	Sjogren’s syndrome	Wedge resection
Kawashima et al. [7]	75/F	None	Multiple	ITP	Right middle lower lobectomy + S3 segmentectomy
Upadhaya et al. [8]	78/F	Dyspnea	Multiple	None	※None
The present case	67/F	None	Multiple	Sjogren’s syndrome	Wedge resection

*RA* rheumatoid arthritis, *ITP* idiopathic thrombocytopenic purpura

^※^Only transbronchial biopsy was performed

Surgical excision and radiotherapy have been sometimes performed as a treatment for primary lung MALT lymphoma; however, chemotherapy is mainly used in multiple lesions and recurrent cases. Chemotherapy with rituximab and cyclophosphamide is recommended [12].

Treatment of pulmonary amyloidosis is not necessary in asymptomatic patients. However, bronchial obstruction or hemoptysis may be treated with laser therapy, and oral steroids may be used to alleviate symptoms. When performing surgical resection, attention should be paid to intraoperative bleeding owing to hypervascular tissues in pulmonary amyloidosis [13].

## Conclusion

In patients with autoimmune disease, when pulmonary amyloid nodules are detected, MALT lymphoma should be considered as a coexisting disease.

## Data Availability

Please contact the author for data requests.
